# A comprehensive comparison of comparative RNA structure prediction approaches

**DOI:** 10.1186/1471-2105-5-140

**Published:** 2004-09-30

**Authors:** Paul P Gardner, Robert Giegerich

**Affiliations:** 1 Department of Evolutionary Biology, University of Copenhagen, Universitetsparken 15, 2100 Copenhagen Ø, Denmark; 2 Faculty of Technology, University of Bielefeld, PO Box 10 01 31, 33501 Bielefeld, Germany

## Abstract

**Background:**

An increasing number of researchers have released novel RNA structure analysis and prediction algorithms for comparative approaches to structure prediction. Yet, independent benchmarking of these algorithms is rarely performed as is now common practice for protein-folding, gene-finding and multiple-sequence-alignment algorithms.

**Results:**

Here we evaluate a number of RNA folding algorithms using reliable RNA data-sets and compare their relative performance.

**Conclusions:**

We conclude that comparative data can enhance structure prediction but structure-prediction-algorithms vary widely in terms of both sensitivity and selectivity across different lengths and homologies. Furthermore, we outline some directions for future research.

## Background

### Motivation

RNA, once considered a passive carrier of genetic information, is now known to play a more active role in nature. Many recently discovered RNAs are catalytic, for example RNase P which is involved in tRNA maturation and the self-splicing introns involved in mRNA maturation [1]. In addition, there is evidence that RNA based organisms were an essential step in the evolution of modern DNA-protein based organisms [2,3]. The number of non-coding RNAs (ncRNA) in humans remains a mystery, but progress in this direction suggests the number of ncRNAs produced is comparable to the number of proteins [4-6]. Surprisingly, the number of protein coding genes does not correlate with our concept of "organism complexity", hence it has been hypothesised that control of gene expression via a combination of alternative splicing and non-coding RNAs are responsible for this, implying that the "Central Dogma" (RNA is transcribed from DNA and translated into protein) at least in higher eukaryotes is woefully inadequate [7,8].

**Figure 1:**
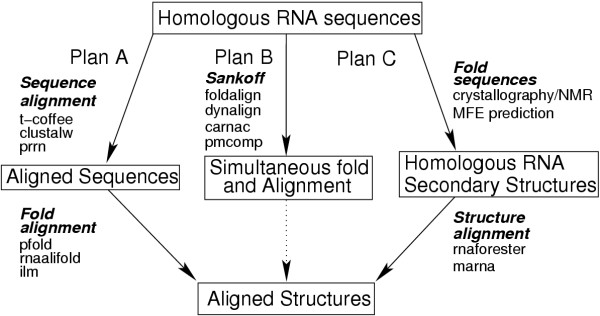
**RNA analysis. **Current automated approaches to analysing homologous RNA sequences and structures usually follow one of three "plans". Plan A uses aligned sequences (usually produced by a standard multiple sequence alignment algorithm) to infer a consensus secondary structure from the evolutionary and energetic information contained in an alignment. This is a highly successful approach, but is limited to data-sets with sequence homology high enough for the alignment step to work yet divergent enough for detection of structurally consistent mutations. Plan B employs the "Sankoff algorithm" to simultaneously align and infer a consensus structure. This algorithm requires extreme amounts of memory and time. Plan C aligns RNA structures rather than sequences. This approach can be used in the rare situation where reliable structures are known. Representative algorithms which could be used for each plan are indicated within the figure.

A fundamental tenet of biology is that a stable tertiary structure is essential for biological function. In the case of RNA the secondary structure (the base-pair set for an RNA molecule) provides a scaffold for the tertiary structure [9,10]. Yet, the experimental determination of RNA structure remains difficult [11]; Researchers increasingly turn to computational methods. To date the most popular structure prediction algorithm is the Minimum Free Energy (MFE) method for folding a single sequence, this has been implemented by two packages: Mfold [12] and RNAfold [13]. However, there are several independent reasons why the accuracy of MFE structure prediction is limited in practise (see discussion below). Generally the best accuracy can be achieved by employing comparative methods [14]. This paper explores the extent to which this statement is true, given the current state of the art, for automated methods. There are currently three approaches to automated comparative RNA sequence analysis where the comparative study is supported by available algorithms (see plans A, B, and C, figure 1). A researcher following plan A may align sequences using standard multiple sequence alignment tools (i.e. ClustalW [15], t-coffee [16], prrn [17],...), then use signals provided by structure neutral mutations for the inference of a consensus structure. Frequently the mutual-information measure is used for this [18-20]. Recently tools have been developed that use a combination of MFE and a covariation-score [21,22] or probabilistic models compiled from large reference data-sets [23,24]. However, a multiple-sequence-alignment step assumes a well conserved sequence. This is often not so with swiftly evolving ncRNA sequences, in this case incorrect sequence alignments can destroy any covariation signal.

This has motivated plan B, the use of the "Sankoff-Algorithm", an algorithm designed for the simultaneous alignment, folding and inference of a protosequence for a set of homologous structural RNA sequences [25]. The recurrences combine sequence alignment and Nussinov (maximal pairing) folding [26]. The algorithm requires extreme computational resources (*O*(*n*^3*m*^) in time, and *O*(*n*^2*m*^) in space, where *n *is the sequence length and *m *is the number of sequences). Current implementations, Foldalign [27,28], Dynalign [29] and PMcomp [26], are restricted implementations of the Sankoff-algorithm which impose pragmatic limits on the size or shape of substructures.

The final approach (plan C) applies when no helpful level of sequence conservation is observed. We may exclude the sequence alignment step, predict secondary structures for each sequence (or sub-group of sequences) separately, and directly align the structures. Because of the nested branching nature of RNA structures, these are adequately represented as trees. The concept of a similarity measurement via edit operations, a standard procedure for string comparisons, has been generalised to trees [30-33]. Tree comparison and tree alignment models have been proposed [34,35] and implemented [13,36-39]. The crucial point in plan C is the question whether the initial independent folding produces at least some structures that align well and hence give clues as to the underlying consensus structure – when one exists. An increasing number of researchers have recently released novel RNA structure analysis and prediction algorithms [22,23,37,40-43]. Yet few algorithms are tested upon standardised example data-sets, and often they are not compared with algorithms of the same pedigree. Algorithm evaluation is a regular event for protein structure prediction groups [44-47], gene-prediction [48-50] and multiple sequence alignments [51-54]. Based on reliable data-sets, we evaluate:

• the viability of plan A, B, or C given tools available today, and

• the relative performance of the tools used within each plan.

We shall explicitly not evaluate computational efficiency, which (by necessity) differs widely between the tools. We also do not evaluate user friendliness (such as ease of installation and convenience of input or output formats, etc.) except for some remarks in the discussion section. Data-sets, documentation and relevant scripts are freely available from .

### Structural alignments and consensus structures

RNA secondary structure inference is the prediction of base-pairs which form the *in vivo *structure, given only the sequence of bases. Three general considerations apply: (1) The *in vivo *structure is not only predetermined by the primary structure, but also by cellular components such as chaperones, base modifications, and even by the transcriptional process itself. There are currently no computational tools available that assess these effects. (2) There are 'ribo-switches', whereby two or more functional structures exist for a given sequence [55-57]. Such cases will fool all the tools studied here, because asking for a single consensus structure is simply the wrong question. On the other hand, the potential of conformational switching can be reliably detected [58-60]. (3) Structures may contain pseudo-knots, which are ignored by most current tools due to reasons of computational complexity and scarcity of these motifs. We do not consider pseudoknots here. However, several comparative approaches that include pseudoknots are currently under development, and certainly merit a comparative study of their own. Note that in an application scenario, we often do not know whether the considerations (1–3) apply.

The comparative approach to structure inference is initiated from a set of homologous RNA sequences. Attempts are made to infer the *in-vivo *structure for each of them, as well as a consensus structure that captures the common, relevant structural aspects. The consensus structure per se does not exist *in vivo, *and so some mathematical rigour should be applied when working with this notion.

An RNA sequence is a string over the RNA alphabet {*A*, *C*, *G*, *U*}. An RNA sequence *B *= *b*_1_,...,*b*_*n *_contains *n *bases, but no structural information. For comparative analysis, we are given the RNA sequences *B*^1^,...,*B*^*k*^. A secondary structure can be associated with each sequence *B *as a string *S *over the alphabet {"(",".",")"}, where parentheses in *S *must be properly nested, and *B *and *S *must be *compatible*: If (*s*_*i*_, *s*_*j*_) are matching parentheses, then (*b*_*i*_, *b*_*j*_) must be a legal base-pair. A base-pair is also denoted as *b*_*i*_·*b*_*j*_, *s*_*i*_·*s*_*j*_, or simply *i*·*j *when the sequence is clear from the context. Both sequences and structures may be padded with a gap symbol "-", in order to align sequences and structures of different lengths. For compatibility of padded sequences and structures, we require that *b*_*i *_= "-" iff s_*i *_= "-".

**Figure 2:**
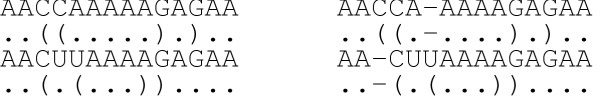
**Alignment consistency. **A violation of RNA structural alignment consistency is shown (left), together with a possible correction (right) – see text for details. Note that the inconsistent alignment may maximise sequence similarity, showing 3 mismatches versus 1 mismatch and 2 indels, with the concrete outcome depending on the gap scoring used. Inconsistency is the reason why it is dangerous to align two *structures *in string representation by a standard *sequence *alignment algorithm. Inconsistency is hard to detect by human eye inspection, and structural alignments in databases are not always free from consistency violations.

A multiple *structural *alignment is a multiple sequence alignment of the 2 * *k *sequences, *B*^1^*, S*^1^,..., *B*^*k*^, *S*^*k*^, such that *B*^*i *^is compatible with *S*_*i*_, and the following *consistency criterion *is satisfied: For any *S*^*i *^and *S*^*j *^and any base-pair 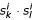, we have 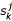 ≠ ")" and 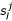 ≠ "(", and if 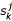 = "(" or 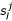 = ")", then 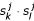. This means that if one partner of a base-pair in *S*^*j *^is aligned to one partner in *S*^*i*^, their partners must also be aligned to each other (see figure 2 for an illustration).

A *consensus structure C *for a multiple structural alignment can be determined by a majority rule approach using a threshold *p *with 0.5 <*p *≤ 1. We define *c*_*k *_= *x *if 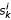 = *x *for at least 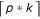 sequences S^*i*^, and *c*_*k *_= ".", otherwise. The latter definition is somewhat arbitrary; when relating the consensus structure to a particular sequence *B *in the alignment, we quietly turn those dots into gaps that align with gaps in *B*. For *p *= 1, we speak of a strict consensus, and the base-pair set in *C *is the intersection of the base-pairs in all *S*^*i*^.

A consensus structure exhibits base-pairs shared by the majority of structures under consideration, but has no sequence information associated with it. Each individual structure for a concrete sequence typically has additional base-pairs which are properly nested between those that constitute the consensus. Given a consensus structure *C *and a sequence *B *compatible with it, we can obtain a structure *refold*(*B*, *C*) which is the best thermodynamic folding for *B *that exhibits the base-pairs specified by *C*, plus additional ones that do not conflict with the former. Refolding can be achieved by *RNAfold *with option -*C *(this option is used to constrain the minimum free energy prediction with prior knowledge – such as known base-pairs, unpaired regions, etc). If *B *and *S *contain gaps, we remove them before refolding and reintroduce them in the same positions afterwards.

Given a consistent structural alignment, it is easy to derive a consensus structure, as we can count majorities at individual positions. If the 5' partner of a base-pair passes the majority threshold, consistency implies that its 3' partner also makes it into the consensus.

Given a consensus structure and a sequence alignment *without *structural information, we can approximate a structural alignment by computing *S*^*i *^= *refold*(*B*^*i*^, *C*). We call this structural alignment reconstruction. While all *S*^*i *^will be consistent with *C*, and with each other as far as the base-pairs of *C *are concerned, they may be inconsistent for the base-pairs introduced in refolding. This is tolerable, since if we trust the consensus to capture the relevant common structural features, there is no need to require that all members of a family agree upon extra-consensus features.

We note in passing that it seems worthwhile to study the conditions under which consensus derivation and structural alignment reconstruction are mutually inverse operations, but such theoretical issues are outside our present scope.

### Interpreting database information

While the plans A, B and C we are about to evaluate strive to find a good consensus structure from sequence data, the "truth" available to us comes in a different form. Structural databases only convey a *consensus by example*: They provide a reference sequence, say *B*^1^, with an experimentally proved structure S^1^, and provide a multiple sequence alignment of B^1^, *S*^1 ^and additional sequences B^2^,..., *B*^*n *^in the family under consideration. The sequence alignment is chosen to exhibit structural similarities between the reference structure and the other family members, but in general, we do not know the precise model of achieving similarity, nor do we know whether this model has been solved to optimality.

One consequence of this situation would be to conclude that the reference structure is the only reliable anchor point available to us for evaluation. Comparative analysis tools would then be evaluated by the capacity to predict this particular structure by using family information. This would be a meaningful way to proceed, however, the effect of structural homogeneity within a sequence family would go unmeasured, and so would the difficulty or success of exploiting it. We therefore proceed in a different way which we call *consensus reconstruction*.

The reference structure *S*^1 ^need not be compatible with any *B*^*i *^except for *i *= 1. However, we can still compute *S*^*i *^:= *refold*(*B*^*i*^, *S*^1^) by treating bases as unpaired where they violate compatibility. (This is also achieved with *RNAfold*, option -C.) What we obtain in this way is a reconstructed structural alignment, which will be consistent to the extent that the reference structure indeed describes the common structural features, and to the extent that the database sequence alignment reflects these. In all our test cases, this alignment was overall consistent, an indicator that the families and their structural features are in fact well defined. From this alignment, we derive a consensus structure as explained above using a threshold *p *= 0.5, which will serve as the standard of truth in our evaluation.

One may argue that our approach to reconstruct the truth is somewhat ad-hoc and should be replaced by a more systematic method. However, this is what the tools we evaluate try to achieve, and we should not add one of our own as the standard of truth. Hence, our consensus reconstruction is designed to stay as close as possible to the database information.

### Caveats

Results of observations based on the above measures must be interpreted with care. We list a number of caveats that must be kept in mind when proceeding to the subsequent sections.

#### Use of defaults

In all tests, one could possibly obtain better predictions by tuning the program's parameters. We felt that it would be inappropriate to do so, since in the evaluation, we know the correct result and could use this knowledge in the tuning, whereas in a true application context, one does not have such guidance. Hence we used the recommended defaults in all cases.

#### Tool abuse

In some cases we apply a tool to data where we know that the model structure has features not recognised by the tool. An example is a structure with multiloops or pseudoknots, searched for with a tool that explicitly excludes such structures. We permit such cases, because again, in a true application context one does not know whether the tool is appropriate or not, and it is still of interest to see how close to the correct structure one can get.

#### Standard of truth

We take for granted the correctness of structural alignments taken from the literature, and the consensus reconstructed thereof. Should one of the tested algorithms produce a result that is actually better (closer to the functionally important structure), it may be penalised. Also, we do not consider a large number of data-sets here, it is possible that performance of some algorithms improves on a different selection of data-sets.

#### Tools improve

Our data reflect the state of the art in 2004. Most of the tools tested are very recent, and their authors are still improving them. Hence, not all observations will remain reproducible. In fact, we hope this study helps to obtain better results in the future.

## Methods

We have compiled RNA sequence alignments consisting of up to 11 sequences derived from reliable sources (see table 1). These have been used to test several RNA analysis packages. Each alignment contains at least one reference sequence *B*^1 ^with (preferably) an experimentally verified secondary structure *S*^1^. Experimental verification of a structure may be from a variety of sources: x-ray crystallography, NMR, enzymatic structure probing or phylogenetic inference. A comparison of phylogenetic with x-ray crystallographic structures has shown the phylogenetic predictions of rRNA to be very reliable (sensitivity > 97%) [61]. This data specifies a "consensus by example", as explained above, to which our consensus reconstruction was applied to obtain the "true" consensus.

To avoid results bias, we constructed test alignments, with corresponding phylogenies that, wherever possible, were free of highly similar clades. In addition, we endeavoured to ensure that the reference sequence was central to the phylogeny, or more specifically, not an out group. To meet these requirements, sequences from large data-sets were sorted into high-similarity and medium-similarity groups (with respect to the model sequence), from which maximum-likelihood phylogenies [62] were constructed. These were pruned until the desired size and topology was achieved. For each data-set two sequence alignments were constructed, one of high sequence identity (approximately 90–99%) and the other more diverse data-set of medium sequence identity (approximately 70–90%).

Our data-sets are quite diverse and must for the purposes of this study be considered difficult to analyse in structural terms. The shape of ribosomal RNA is believed to be influenced by interaction with ribosomal proteins. The shape of RNase P shows relatively little sequence and structure conservation, and furthermore, it contains pseudoknots which are generally excluded by prediction algorithms. Transfer RNAs are known to be a hard case for thermodynamic folding, primarily due to the propensity of modified bases which influence structure formation. All tools tested may perform better upon less complex data-sets, but the purpose of this study is not to show how good the algorithms are but to compare relative performance when prediction is difficult.

### Performance Measures

*Sensitivity *(*X*) and *selectivity *(*Y*) are common measures for determining the accuracy of prediction methods [63]. Selectivity is also known as the "specificity" [28] and "positive predictive value" [64,65]. We use slightly modify versions of the standard definitions of *X *and *Y *for examining RNA secondary structure prediction:


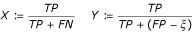


where *TP *is the number of "true positives" (correctly predicted base-pairs), *FN *is the number of "false negatives" (base-pairs in the reference structure that were not predicted) and *FP *is the number of "false positives" (in-correctly predicted base-pairs). However, not all *FP *base-pairs are equally false! We classify *FP *base-pairs as either *inconsistent*, *contradicting *or *compatible. *Predicted base-pairs which conflict with a base-pair in the reference structure are labelled *inconsistent *(i.e. *i*·*j *is predicted where either *i*·*k *and/or *h*·*j *are paired in the reference structure (*h *≠ *i *and *j *≠ *k*)). Predicted base-pairs (*i*·*j*) which are non-nested with respect to the reference structure are labelled *contradicting *(i.e. there exists base-pairs *k*·*l *in the reference satisfying *k *<*i *<*l *<*j*). Note that some base-pairs may both contradict and be inconsistent with the reference structure. Predicted base-pairs which are neither true positive, contradicting or inconsistent are labelled *compatible *and can be considered neutral with respect to algorithm accuracy. Hence these are subtracted in the selectivity evaluation, their number is *ξ *in the above equation. It is of interest to note that the base-pair metric [66,67] between the reference and predicted structures *d*_*BP*_(*S*_*ref*_, *S*_*pred*_) is the sum of *FN *and *FP*, and hence is different from the measure used here.

A measure combining both selectivity and sensitivity is useful for ranking algorithms. For this we employ the *Matthews correlation coefficient *[63] defined below:


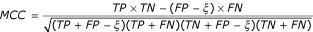


*MCC *ranges from -1 for extremely inaccurate (*TP *= *TN *= 0) to 1 for very accurate predictions (*FP *- *ξ *= *FN *= 0). When comparing RNA structures *TN *= 0 occurs only in extreme examples, hence *MCC *generally ranges from 0 to 1. Furthermore, for the specific case of RNA structure comparisons, *MCC *can be approximated by the arithmetic-mean or geometric-mean of *X *and *Y *[28].

## Results

### Single sequence methods

The accuracy of the MFE single sequence method has been evaluated elsewhere and was found to have an accuracy of 73% when averaged over many different RNAs and "base-pair slippage" was tolerated in the evaluation [68]. A recent and more stringent work found MFE predictions had a sensitivity of 56% and selectivity of 46% for RNase P, SRP and tmRNA structures [64]. Similar values are also reported by the "Gutell Lab" for tRNA and rRNA structures [69-71]. We need to clarify the accuracy of this method on the particular data-sets we employ here for comparison with the multi-sequence methods. After all, if MFE folding worked perfectly for our given data-sets, there would be no need to resort to comparative methods.

### Mfold & RNAfold

Mfold [12,72] and RNAfold [13,73] both implement the Zuker-Stiegler algorithm for computing minimal free energy (MFE) structures assuming a "nearest neighbour model" and using empirical estimates of thermodynamic parameters for neighbouring interactions and loop entropies to score structures. The algorithm is *O*(*n*^3^) in time and *O*(*n*^2^) in memory where *n *is the sequence length. Both employ the same thermodynamic parameters [68]. Hence, differences in the predictions are generally minor and are the result of slightly different implementations. There appears to be no significant differences in terms of algorithm accuracy.

**Figure 3:**
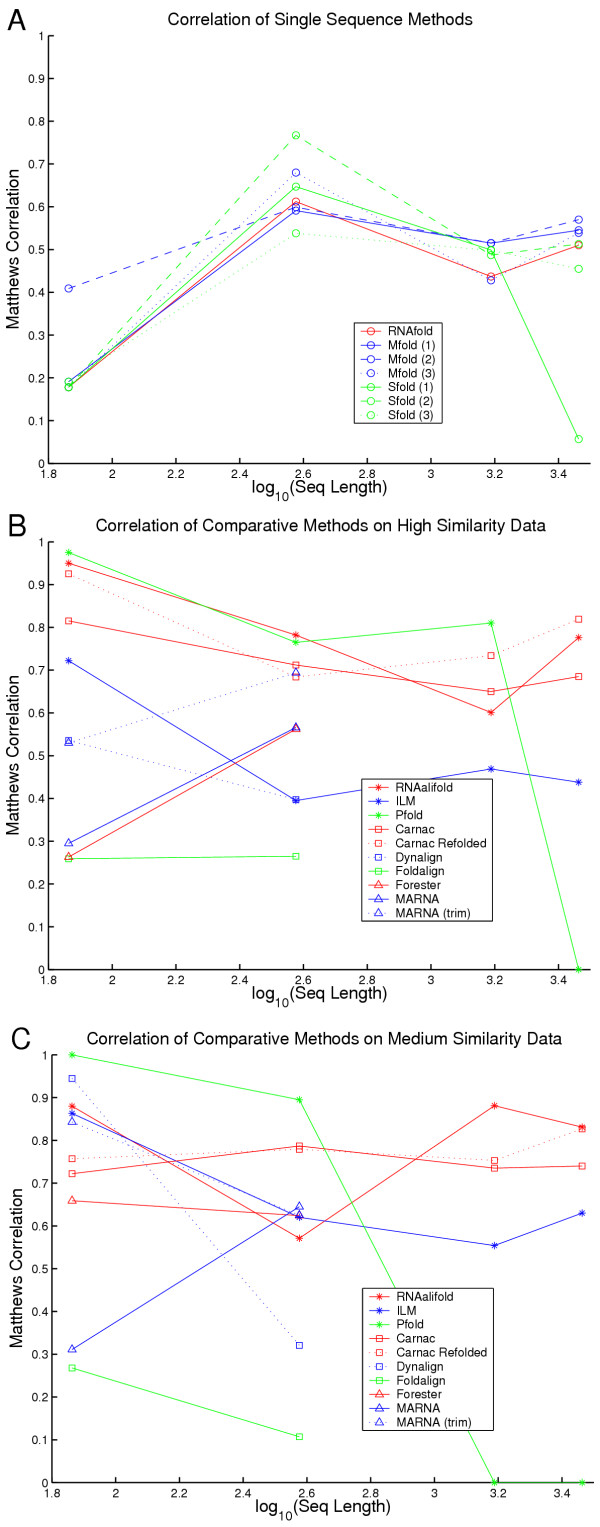
**Prediction correlation with reality. **Matthews correlation coefficient versus the logarithm of the sequence length for a range of different ncRNAs and structure prediction algorithms. Inset **A **shows accuracies of thermodynamic single sequence prediction algorithms. Insets **B **and **C **shows accuracies of comparative methods on the high and medium similarity data-sets respectively.

**Figure 4:**
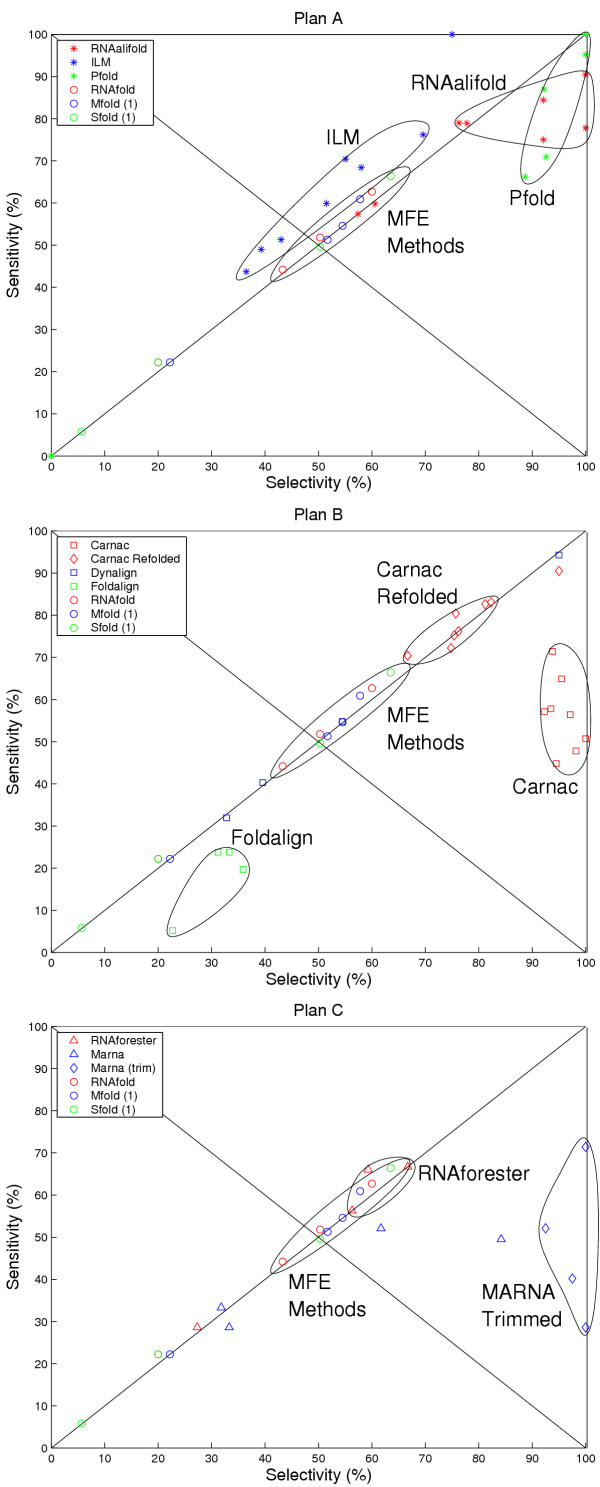
**ROC plots. **We use ROC (receiver operating characteristic) plots to simultaneously display both sensitivity and selectivity for plans A, B and C respectively. Accuracies of the MFE methods (MFold, RNAFold and SFold) are shown in each plot to provide a base-line. Points on the line *X *= *Y *are as sensitive as they are selective, points below this line indicates a greater selectivity, points above indicate greater sensitivity. Points below the line *X *= 100 - *Y *are worse than "random" assignments; Assuming base-pairs are independent of each other (this is false for base-pairing). Points in the top right corner are "perfect" predictions. Interestingly many algorithms form characteristic clusters in these plots. Where the variance is sufficiently small these have been indicated with a closed curve.

The sensitivity, selectivity and correlation of MFE methods (for the four data-sets considered here) ranged from 22–63%, 20–60% and 0.18–0.61 respectively (See figures 3 &4). The low accuracies (22%, 20% & 0.18) are due to an alternative long-stem conformation of *S. cerevisiae *tRNA-PHE which the free energy methods favour. Mfold infers 'suboptimal' structures by calculating minimum free energy structures with the restriction that every possible base-pair is forced in a one-by-one fashion. Unique structures are then ranked by energy. Investigating the top two suboptimal structures from Mfold resulted in an overall increase in the range of sensitivity, selectivity and correlation, 22–69%, 20–67% and 0.18–0.68 respectively. The predictions shown here are used to illustrate the potential advantages of using comparative analyses over single sequence methods.

### Sfold

Sfold [41,74] represents another energy-based single-sequence folding algorithm. For a given RNA, Sfold stochastically samples all possible structures in the Boltzmann ensemble of secondary structures using conditional probabilities which are computed with the partition function [75]. Clustering techniques could then be used to obtain representative ' likely ' structures. Instead, the current implementation samples 1000 structures, sorts these by energy, the minimum and maximum energy structures are computed and the energy range divided into 10 equally sized energy blocks. The minimum energy structure from each block is returned with ranking 1 to 10. We consider the top 3 structures labelled 'Sfold (1–3)'. In terms of accuracy, the results are very similar to those of the Zuker-Stiegler single sequence methods, although with a slightly higher variance (See figures 3 &4).

### Intrinsic limits of single sequence methods

There are systematic limits to the accuracy of single sequence prediction methods. The thermodynamics may not be accurate, as some parameters are extrapolated and parameter measuring conditions *in vitro *are different from *in vivo *conditions. Indeed the thermodynamic model itself is an estimate of the real physics of RNA folding. Also, many bases of structural RNAs are chemically modified by sugar methylation, pseudo-uridine, dihydrouracil, etc, these are generally ignored by these methods. Kinetics of folding are also ignored. Given only a single sequence, we have no way to distinguish base-pairs and structure elements important for the consensus from those that are peculiar for the given sequence. Finally, some functional RNAs have bistable structures, while in others, the structure is irrelevant, hence not conserved, and the optimal MFE structure is biologically meaningless. This is some justification of why researchers proceed to comparative methods.

### Comparative method: alignment folding (plan A)

To simulate realistic RNA folding studies we use ClustalW [15] to re-align each of our test data-sets, then folded these using each of the methods mentioned below. The resultant predicted structures were then compared to our reconstructed consensus structures.

#### RNAalifold

RNAalifold [21,76] implements an extension of the Zuker-Stiegler algorithm for computing a consensus structure from RNA alignments. The algorithm computes an averaged energy matrix 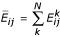 (where *N *is the number of sequences in the alignment) and a covariation score matrix, augmented with penalties for inconsistent sequences, *B*_*ij*_. A standard trace-back procedure is performed to recover a consensus structure with the optimal sum-of-average-energy-and-covariation-score 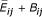. The algorithm is remarkably efficient *O*(*N*·*n*^2 ^+ *n*^3^) in time and *O*(*n*^2^) in memory.

The sensitivity, selectivity and correlation of the RNAalifold predictions ranged from 57–91%, 57–100% and 0.57–0.95 respectively, showing a significant increase in the accuracy measures when compared to the MFE-methods.

#### Pfold

Pfold implements a "stochastic context free grammar" (SCFG) designed to produce a "prior probability distribution of RNA structures" for an RNA alignment input [23,24,77]. A maximum-likelihood phylogeny is used to weight posterior probabilities computed from large reference data-sets.

The algorithm is generally accurate and efficient. Hence, the over-all sensitivity, selectivity and correlation of the Pfold predictions ranged from 0–100%, 0–100% and 0.0–1.0, respectively. But removing those points where Pfold predictions were empty structures (LSU rRNA (H & M) and SSU rRNA (M), see figure 3), the prediction accuracies ranged from 66–100%, 89–100% and 0.77–1.0, respectively. The zeros are due to 'under-flow errors', a solution is presently under construction by the authors (pers. commun. Bjarne Knudsen).

#### ILM

ILM (iterated loop matching) is one of the few comparative RNA folding algorithms which can return pseudo-knotted structures [22,78]. It uses a combination of thermodynamic and mutual information content scores [18] to produce a secondary structure. All possible stems ("small" internal loops and bulges inclusive) are generated and ranked according to a combination of thermodynamic and mutual-information scores. The stem with maximal score is selected, scores are updated and stems conflicting the selection removed, then the next highest scoring stem is selected. This algorithm is iterated until no stems remain. ILM generally ranked low in terms of selectivity and was not as sensitive as either RNAalifold or Pfold on the high similarity data, but did improve on the medium similarity data-sets (see figure 3). The over-all sensitivity, selectivity and correlation of ILM predictions ranged from 44–100%, 37–75% and 0.40–0.86, respectively. To ensure the low selectivity values weren't due to the reference-structure being pseudo-knot free we re-evaluated ILM with reference-structures replete with pseudo-knots. The new sensitivity, selectivity and correlation values ranged from 31–100%, 26–75% and 0.29–0.86, in fact evaluating with pseudo-knotted structures did little to increase ILM selectivity. But, keep in mind that the sensitivity of the other (non-knot-inclusive) methods *must *decrease when a significant proportion of the true base-pairs are engaged in pseudo-knots.

The inclusion of pseudo-knots prediction vastly increases the number of possible secondary structures, this is why they are generally excluded from exhaustive folding algorithms. In addition, there is a general lack of experimentally derived thermodynamic parameters which include pseudo-knots. ILM is a method still under development, hence the performance may improve once pseudo-knots can be more accurately modelled.

### Comparative method: simultaneous sequence alignment and folding (plan B)

#### Sankoff

The Sankoff algorithm is a dynamic programming approach to obtain a common base-pair list with maximal sum of base-pair weights. Basically, this is a merger of sequence alignment and Nussinov [79] (maximal-pairing) folding dynamic programming methods [26]. Sankoff's algorithm can be used to obtain both an alignment and consensus structure. Full implementations of the "Sankoff algorithm" for the solution of simultaneous RNA folding, alignment and protosequence problems have proven too computationally taxing (*O*(*n*^3*m*^) in time, and *O*(*n*^2*m*^) in space for sequence length *n *and *m *sequences) to be practical [25]. Hence, three restricted versions of this algorithm have been implemented. These are Foldalign [27], Dynalign [29] and recently PMcomp has also been published [26]. Carnac [80,81] is another recent innovation designed to detect conserved stems in unaligned sequences, we include it here as a relative of the Sankoff approach.

#### Foldalign

Foldalign [27] can be interpreted as "a mixture of local alignment and maximum number of base-pairs algorithm" [28,82]. A combination of "clustal" [15] and "consensus" [83] heuristics are used to build multiple sequence alignments from pair-wise comparisons. Restricting maximum motif size (for this study 50 was used) and forbidding bifurcating structures (multi-loops) reduces the time complexity to *O*(*n*^4^*N*) in time (where *N *is the number of sequences and *n *is the length of the longest sequence). A simple match-based scoring scheme is used to rank putative conserved structure elements.

The Tool Abuse Caveat generally applies to the tool Foldalign as all of our data-sets contain multi-loops. The use of Foldalign for the prediction of global, multi-looped secondary structures is not recommended-as Foldalign is specifically designed for the location of short regulatory motifs such as IREs [84] where the motifs are only related at the level of (non-bifurcating) structure and not at the level of sequence. Hence the relatively poor sensitivity, selectivity and correlation, which ranged from 5–24%, 23–36% and 0.11–0.27 respectively, for our test data-sets.

#### Dynalign

Dynalign [29,85] is a pairwise implementation of the Sankoff algorithm, which uses a "full energy model" to locate a common low energy structure (including multi-loops) and align two structural RNAs. The computational complexity of the full Sankoff is reduced by restricting the difference in the indices *i *and *j *of aligned nucleotides (where *i *indexes positions in sequence 1 and *j *indexes sequence 2) to be less than *M*. In addition, Dynalign uses the same method employed by MFold to reduce the conformation space, by limiting the size of internal loops [29,86]. The complexity is thus reduced to *O*(*n*^3^*M*^3^).

The current Dynalign implementation is restricted to pair-wise sequence comparisons. Rather than compute all 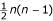 pairwise foldings we compared all sequences with the reference structure. Due to the computational expense of this algorithm it could only be used to predict tRNA and RNase P structures. Dynalign performed well on the tRNA, medium sequence homology data-set (sensitivity, selectivity and correlation of 94%, 95% and 0.94 respectively, when averaged over all pairwise alignments with the reference sequence). With this one high-scoring point removed, averaged sensitivity, selectivity and correlation values ranged from 32–54%, 33–54% and 0.32–0.54 respectively. Comparing the performances of MFold and Dynalign showed that MFold performance was always superior on the RNase P data-set, Dynalign however did much better on the shorter and more diverse tRNA sequences. Performance gains could be made by investing more computer time and refolding RNase P with larger ' maximum insert size', which was set to 10 during this study. The use of Dynalign on the RNase P data-sets in this study is therefore a case of tool-abuse, as the parameters recommended by the authors of Dynalign were not used (to ensure calculations completed in reasonable time).

#### Carnac

The Carnac algorithm, as mentioned previously, is not strictly an implementation of the Sankoff algorithm. A set of filters are employed through which sets of sequences are passed in a pair-wise fashion [80,81,87]. Sequences are scanned for stems and "high similarity" regions of sequences (dubbed "anchor points") are identified, a dynamic program is used to select conserved stems using anchor point and covariation information.

The Carnac algorithm was remarkably selective at base-pair predictions. However, the sensitivity of the algorithm was generally low, although when evaluated with the correlation coefficient it is comparable to RNAalifold and Pfold. Sensitivity, selectivity and correlation values for Carnac predictions ranged from 45–71%, 92–100% and 0.65–0.82 respectively. The sensitivity of Carnac can be increased by constraining a minimum free energy fold (i.e. with "RNAfold-C") with the Carnac predicted structure, but this cost in terms of selectivity. On average this increased the sensitivity by 22.5, decreased the selectivity by 17.2 and slightly increased the correlation by 0.05.

### Alignment of predicted structures (plan C)

#### RNA forester

RNAforester [37,88] implements the tree alignment model. In contrast to approaches that produce only a similarity value, but no underlying alignment, it computes pairwise alignments of two input structures. RNAforester can produce either global or local alignments; we used the global mode. A structure alignment is itself a branching (tree-like) structure; the set of matched base-pairs can be derived from it and evaluated as with the other approaches.

We used the tRNA and RNase P data-sets and generated structure single sequence predictions with RNAfold. All predicted structures were aligned pairwise and a neighbour-joining approach used to cluster and align high similarity sequences and structure profiles. The highest scoring alignment was used to derive a predicted consensus that was evaluated against the consensus tRNA model structures. Sensitivity, selectivity and correlation ranges of consensus structures computed from the highest scoring RNAforester alignments were 29–67%, 27–67% and 0.26–0.66 respectively. It seems likely that much of the inaccuracy of this approach is due to MFE structure prediction, however the structure-clustering approach frequently separates mis-folded MFE predictions from the accurate folds.

#### MARNA

The MARNA algorithm [39,89] proceeds by constructing edge weights between nucleotides in a pairwise fashion. Weights are structure-enhanced-sequence-similarities transformed from edit distances proposed by Zhang [90]. Phase two pipes the set of alignment edges into t-coffee [16] for multiple alignment production. The resultant alignments are not strictly structural alignments in the sense defined above. Rather, these are sequence alignments influenced by structure.

Sensitivity, selectivity and correlation values of consensus structures computed from MARNA alignments of MFE structures ranged from 29–52%, 32–84% and 0.30–0.65 respectively. We also tried trimming high entropy base-pairs from the MFE predictions using the bound *Q*_*ij *_> 1, where 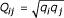, 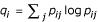, and *p*_*ij *_are pair-probabilities computed using McCaskilPs partition function [75]. The new accuracy ranges were 29–71%, 92–100% and 0.53–0.84. A related approach for trimming of low probability was recently shown to improve the selectivity of MFE predictions [65]. MARNA is generally less dependant upon the accuracy of the input structures hence performs slightly better with the poorly predicted tRNA structures than RNAforester.

## Discussion

We have evaluated three different strategies for comparative structure prediction, and altogether eight tools (not counting the single sequence methods). The results of which are summarised in figures 3 &4. A surprising discovery given that the test data-sets are so diverse is that algorithm specific clusters formed in sensitivity versus selectivity scatter plots, indicating algorithm-specific eccentricities. A number of algorithms which might have been evaluated here have been excluded, primarily due to the heavy computational costs of the various implementations on our longer data-sets. We favoured recent algorithms which could be compiled on modern computers and those with input and output which could be simply dealt with (for example returning dot-bracket [13,37,91] or tabular-connect type formats [12,29,41], rather than coordinates and lengths of stacks or graphic (gif/pdf) representations favoured by a minority of researchers).

### Practical recommendations

For well aligned short sequences, both Pfold and RNAalifold generally perform well, PFold performed marginally better than RNAalifold. It is likely that some moderate refinements to RNAalifold would improve accuracy without altering the efficiency, for example, if gaps were not penalised in the free-energy evaluation and a more sophisticated model for scoring mutations was employed, perhaps ribosum matrices [92] could be used to weight base-pair bonuses and penalties. For well aligned, long sequences the performance and speed of RNAalifold was excellent. For data-sets consisting of short (< 200 bases) and diverse sequences Dynalign might do well, as it does not require sequence similarity – in fact the scoring function does not include sequence comparison. Otherwise, one might choose to use a mixture of RNAalifold and/or Pfold to fold similar clades and RNAforester and/or MARNA to align folded clades. Advocates of plan A should note that many multiple sequence alignment algorithms generally do not favour transitions over transversions or employ ad hoc 2-parameter methods to model these (ClustalW [15] for example). Structural RNA sequences however evolve rapidly via structure neutral mutations which are frequently transitions and rarely transversions [92,93]. Multiple sequence algorithms which employ more complex yet more accurate models of sequence evolution will undoubtedly produce "better" alignments for folding.

Carnac produced highly selective structures for all the test data-sets, which if used to constrain a free energy fold produced sensitive predictions with a cost to selectivity. The consistency of Carnac performance is remarkable, for all the data-sets considered here this heuristic approach performed well. It is however unclear how Carnac will perform on highly diverse data-sets.

For advocates of plan C, we have an encouraging message: Both MARNA and RNAforester perform better on the medium similarity data than on high similarity data. This seems paradoxical at first glance, but one must understand that for an approach purely based on predicted structures, high sequence similarity can be a curse rather than a blessing: If sequences are very similar, they may jointly fold into the wrong MFE structure. With more sequence variation, it becomes more likely that at least some family members have good predictions, which by their mutual similarity can be picked out from the rest. This means that especially in the case of low sequence similarity, where nothing else works, plan C, currently the least explored strategy of all, has a certain promise.

## Conclusions

Finally, let us outline some directions for future research.

An implementation of the single sequence pseudoknot algorithms [42,43,94] employing similar strategies to RNAalifold [21] for alignment folding would be most useful. Based upon the RNAalifold results this approach would dramatically increase the accuracy of these algorithms upon certain data-sets. Also, an extension of these allowing constrained foldings to incorporate prior knowledge would be of assistance, this has proved extremely useful for MFE predictions. Sampling structures from reference alignments is also likely to prove beneficial. The implementation of fast and accurate variants of the Sankoff algorithm remains an open problem.

Again allowing constrained foldings and alignments would be useful. The further development of "BLAST-like" folding heuristics for this should be a priority, obviously Carnac is a good start. The MARNA approach for producing structurally enhanced multiple alignments produced rather selective results after trimming high-entropy base-pairs from MFE predictions. This suggests that weighting edit-distances with partition-function derived probabilities or entropies will produce reasonable RNA alignments. A consensus structure could then be derived from MFE-structures or from PFold or RNAalifold predictions on the resultant alignment. This approach would effectively decouple the Sankoff algorithm into manageable structure-enhanced-alignment and folding stages.

### Note added in proof

Two further developments are likely to increase the power of plan C. Pure multiple structure alignment (as opposed to pairwise alignment used here) presented in [95] may leave out some misfolded structures from a progressively constructed profile aligment. A small but representative set of near-optimal structures can now be derived by abstract shape analysis [96]. Combining both approaches, one could consider a progressive multiple alignment approach where these representative, near-optimal structures are included for each sequence.

More training data is essential for this field to progress, for this homology search tools are essential. Infernal [91,97] used to construct the Rfam database [98,99] is an excellent approach but sensitivity might be increased with a phylogenetic approach and RNA-specific sequence search tools. The implementation of methods combining energetics, covariation [21] and co-transcriptional folding [100] in a statistically reasonable manner is also a potentially fruitful direction for development.

## Authors' contributions

PPG carried out the experiments, the analysis and drafted the manuscript. RG suggested comparing comparative structure prediction methods and assisted in the manuscript preparation. All authors read and approved the final manuscript.

## Figures and Tables

**Table 1:** Characteristics and sources of the four test data-sets, columns from left to right show: data-set, lengths, mean pair-wise sequence similarity (mean pair-wise Kimura "2-parameter" distance is shown in parentheses [109]), the number of sequences in each alignment and the alignment and structure sources are given.

		**Test data-set characteristics and sources**					
Data-set	length	mean pairwise seq. identity		Number of Sequences		Alignment source	Structure source

		High	Med.	High	Med.		
*E. coli *LSU rRNA	2904	88.1 (0.12)	72.0 (0.35)	11	11	Wuyts *et al*., (2001)	Cannone *et al*., (2002)
*E. coli *SSU rRNA	1542	90.7 (0.08)	80.0 (0.21)	11	11	Wuyts *et al*., (2002)	Cannone *et al*., (2002)
*E. coli *RNase P	377	81.5 (0.09)	67.1 (0.41)	9	11	Brown, (1999)	Brown, (1999)
*S. cerevisiae *tRNA-PHE	73	84.4 (0.19)	60.0 (0.71)	11	11	Griffiths-Jones *et al*., (2003)	Sundaralingham & Rao, (1975)

**Table 2:** The following tables display results of several structure predictions using a variety of algorithms upon data-sets containing either ***S. cerevisiae *tRNA-PHE, *E. coli *RNase P, *E. coli *SSU rRNA **or ***E. coli *LSU rRNA **sequences. Reading columns from left to right we show: prediction method, number of base-pairs in the reference structure, number of base-pairs in the predicted structure, the number of true positive base-pairs in the prediction (% sensitivity as described earlier in parentheses), the number of false positive base-pairs in the prediction (% selectivity as described earlier in parentheses), correlation values are the "Matthews correlation coefficient" (with approximate correlation in parentheses). Each of these MFE-based attempts to predict the famous *S. cerevisiae *tRNA-PHE structure converges on an alternative lengthy-helix type structure. Adding prior knowledge, such as forcing modified bases in the RNA sequence to be unpaired can produce dramatic improvements.

	***S. cerevisiae *tRNA-PHE: Single Sequence Methods**				
Algorithm	number of bps in reference	number of bps in prediction	True Positives (% sensitivity)	False Positives (% selectivity)	Correlation (%)

RNAfold	18	23	4 (22.2)	16 (20.0)	0.178 (21.1)
Mfold (1)	18	21	4 (22.2)	14 (22.2)	0.191 (22.2)
Mfold (2)	18	22	8 (44.4)	11 (42.1)	0.409 (43.3)
Mfold (3)	18	23	4 (22.2)	16 (20.0)	0.178 (21.1)
Sfold (1)	18	23	4 (22.2)	16 (20.0)	0.178 (21.1)
Sfold (2)	18	23	4 (22.2)	16 (20.0)	0.178 (21.1)
Sfold (3)	18	21	4 (22.2)	14 (22.2)	0.191 (22.2)

**Table 3:** Generally the comparative approaches perform much better than MFE methods at determining *S. cerevisiae *tRNA-PHE structure. For the consensus predictions of RNAalifold and Carnac we also computed "filled" structures using constrained MFE predictions. This usually improved the sensitivity of the methods. PFold a built-in stem-extension procedure to fill structures. As the tRNA structure contains a multi-loop Foldalign is not expected to perform well here. Dynalign performed well on the most diverse data-set (M) but didn't do well on the high similarity data-set. The structure alignment methods generally did poorly here. Most probably due to the miss-folded MFE structure which were used as input. Trimming high entropy base-pairs from the input structures produced modest improvements.

	***S. cerevisiae *tRNA-PHE: Comparative Methods**				
Algorithm	number of bps in reference	number of bps in prediction	True Positives (% sensitivity)	False Positives (% selectivity)	Correlation (%)

Plan A: ClustalW Alignment					
RNAalifold (H)	21	20	19 (90.5)	0 (100.0)	0.950 (95.2)
RNAalifold (H) + RNAfold-C	21	21	21 (100.0)	0 (100.0)	1.000 (100.0)
RNAalifold (M)	18	14	14 (77.8)	0 (100.0)	0.880 (88.9)
RNAalifold (M) + RNAfold-C	18	21	18 (100.0)	0 (100.0)	1.000 (100.0)
ILM (H)	21	24	16 (76.2)	7 (69.6)	0.722 (72.9)
ILM (M)	18	30	18 (100.0)	6 (75.0)	0.863 (87.5)
Pfold (H)	21	21	20 (95.2)	0 (100.0)	0.975 (97.6)
Pfold (M)	18	21	18 (100.0)	0 (100.0)	1.000 (100.0)
Plan B: Unaligned sequences					
Carnac (H)	21	17	15 (71.4)	1 (93.8)	0.815 (82.6)
Carnac (H) + RNAfold-C	21	21	19 (90.5)	1 (95.0)	0.925 (92.7)
Carnac (M)	21	13	12 (57.1)	1 (92.3)	0.722 (74.7)
Carnac (M) + RNAfold-C	21	22	16 (76.2)	5 (76.2)	0.757 (76.2)
Dynalign (H)	21	22.40	11.50 (54.78)	10.20 (54.45)	0.5353 (54.59)
Dynalign (M)	21	21.10	19.80 (94.27)	1.20 (95.00)	0.9448 (94.64)
Foldalign (H)	21	16	5 (23.8)	11 (31.2)	0.259 (27.5)
Foldalign (M)	21	16	5 (23.8)	10 (33.3)	0.268 (28.6)
Plan C: Structure alignment					
MARNA (H)	21	19	6 (28.6)	12 (33.3)	0.295 (31.0)
MARNA (M)	21	22	7 (33.3)	15 (31.8)	0.311 (32.6)
MARNA-trim (H)	21	6	6 (28.6)	0 (100.0)	0.530 (64.3)
MARNA-trim (M)	21	15	15 (71.4)	0 (100.0)	0.843 (85.7)
RNAforester (H)	21	23	6 (28.6)	16 (27.3)	0.263 (27.9)
RNAforester (M)	21	21	14 (66.7)	7 (66.7)	0.659 (66.7)

**Table 4:** Note the improvement in prediction accuracy on the supposedly more difficult and longer *E. coli *RNase P data-set. This shows that MFE methods are less sensitive to folding errors on longer data-sets but are also less likely to resolve the entire structure. There is little difference in algorithm accuracy for each of the methods explored here. Each employs the same energy parameters so differences are due to slightly different implementations.

	***E. coli *RNase P: Single Sequence Methods**				
Algorithm	number of bps in reference	number of bps in prediction	True Positives (% sensitivity)	False Positives (% selectivity)	Correlation (%)

RNAfold	110	116	69 (62.7)	46 (60.0)	0.612 (61.4)
Mfold (1)	110	118	67 (60.9)	49 (57.8)	0.591 (59.3)
Mfold (2)	110	114	67 (60.9)	46 (59.3)	0.599 (60.1)
Mfold (3)	110	118	76 (69.1)	37 (67.3)	0.680 (68.2)
Sfold (1)	110	116	73 (66.4)	42 (63.5)	0.647 (64.9)
Sfold (2)	110	119	86 (78.2)	28 (75.4)	0.767 (76.8)
Sfold (3)	110	117	61 (55.5)	55 (52.6)	0.538 (54.0)

**Table 5:** RNase P is a difficult data-set to study. Five sequences in the high similarity data-set are truncated at both the 5 and 3 prime ends (due to the primers used for sequencing these). Sequences in the medium similarity data-set are full-length but do not align well using traditional tools such as ClustalW. Values corresponding to the re-evaluation of ILM with pseudo-knot inclusive reference structures are indicated by "ILM-pknot".

	***E. coli *RNase P: Comparative Methods**				
Algorithm	number of bps in reference	number of bps in prediction	True Positives (% sensitivity)	False Positives (% selectivity)	Correlation (%)

RNAalifold (H)	71	113	56 (78.9)	16 (77.8)	0.782 (78.3)
RNAalifold (H) + RNAfold-C	71	119	55 (77.5)	16 (77.5)	0.773 (77.5)
RNAalifold (M)	54	66	31 (57.4)	23 (57.4)	0.571 (57.4)
RNAalifold (M) + RNAfold-C	54	77	33 (61.1)	16 (67.3)	0.639 (64.2)
Pfold (H)	71	67	47 (66.2)	6 (88.7)	0.765 (77.4)
Pfold (M)	54	87	47 (87.0)	4 (92.2)	0.895 (89.6)
ILM (H)	71	124	31 (43.7)	54 (36.5)	0.395 (40.1)
ILM (M)	54	133	38 (70.4)	31 (55.1)	0.620 (62.7)
ILM-pknot (H)	110	124	53 (48.2)	65 (44.9)	0.463 (46.5)
ILM-pknot (M)	110	133	44 (40.0)	75 (37.0)	0.382 (38.5)
Plan B: Unaligned sequences					
Carnac (H)	71	40	36 (50.7)	0 (100.0)	0.712 (75.4)
Carnac (H) + RNAfold-C	71	116	50 (70.4)	25 (66.7)	0.684 (68.5)
Carnac (M)	97	80	63 (64.9)	3 (95.5)	0.787 (80.2)
Carnac (M) + RNAfold-C	97	118	78 (80.4)	25 (75.7)	0.779 (78.1)
Foldalign (H)	71	41	14 (19.7)	25 (35.9)	0.265 (27.8)
Foldalign (M)	97	24	5 (5.2)	17 (22.7)	0.107 (13.9)
Dynalign (H)	71	95.13	28.63 (40.31)	41.50 (39.59)	0.3974 (39.96)
Dynalign (M)	97	103.20	31.00 (31.95)	61.50 (32.80)	0.3208 (32.39)
Plan C: Structure alignment					
MARNA (H)	71	89	37 (52.1)	23 (61.7)	0.566 (56.9)
MARNA (M)	97	60	48 (49.5)	9 (84.2)	0.645 (66.8)
MARNA-trim (H)	71	52	37 (52.1)	3 (92.5)	0.694 (72.3)
MARNA-trim (M)	97	43	39 (40.2)	1 (97.5)	0.625 (68.9)
RNAforester (H)	71	114	40 (56.3)	31 (56.3)	0.562 (56.3)
RNAforester (M)	97	117	64 (66.0)	44 (59.3)	0.624 (62.6)

**Table 6:** *E. coli *SSU rRNA with a length of approximately 1600 nucleotides is beyond the reach of many structure prediction algorithms such as RNAforester and Dynalign. The minimum free energy methods, however, can produce results.

	***E. coli *SSU rRNA: Single Sequence Methods**				
Algorithm	number of bps in reference	number of bps in prediction	True Positives (% sensitivity)	False Positives (% selectivity)	Correlation (%)

RNAfold	468	493	207 (44.2)	271 (43.3)	0.437 (43.8)
Mfold (1)	468	480	240 (51.3)	224 (51.7)	0.515 (51.5)
Mfold (2)	468	487	242 (51.7)	229 (51.4)	0.515 (51.5)
Mfold (3)	468	487	202 (43.2)	273 (42.5)	0.428 (42.8)
Sfold (1)	468	481	232 (49.6)	229 (50.3)	0.499 (49.9)
Sfold (2)	468	499	231 (49.4)	249 (48.1)	0.487 (48.7)
Sfold (3)	468	475	232 (49.6)	230 (50.2)	0.498 (49.9)

**Table 7:** The probabilistic approach of PFold can, on occasion, suffer from "under-flow" errors caused by multiplying many probabilities together producing numbers too low to be dealt with on modern computers. This is what has happened on the medium similarity data-set.

	***E. coli *SSU rRNA: Comparative Methods**				
Algorithm	number of bps in reference	number of bps in prediction	True Positives (% sensitivity)	False Positives (% selectivity)	Correlation (%)
Plan A: ClustalW Alignment					
RNAalifold (H)	460	472	275 (59.8)	179 (60.6)	0.601 (60.2)
RNAalifold (H) + RNAfold-C	460	483	273 (59.3)	195 (58.3)	0.588 (58.8)
RNAalifold (M)	441	433	372 (84.4)	32 (92.1)	0.881 (88.2)
RNAalifold (M) + RNAfold-C	441	469	388 (88.0)	44 (89.8)	0.889 (88.9)
Pfold (H)	460	377	326 (70.9)	26 (92.6)	0.810 (81.7)
Pfold (M)	441	0	0 (0.0)	0 (0.0)	0.000 (0.0)
ILM (H)	460	565	236 (51.3)	313 (43.0)	0.469 (47.1)
ILM (M)	441	564	264 (59.9)	249 (51.5)	0.554 (55.7)
ILM-pknot (H)	468	565	236 (50.4)	311 (43.1)	0.466 (46.8)
ILM-pknot (M)	468	564	266 (56.8)	258 (50.8)	0.537 (53.8)
Plan B: Unaligned sequences					
Carnac (H)	460	233	206 (44.8)	12 (94.5)	0.650 (69.6)
Carnac (H) + RNAfold-C	460	470	332 (72.2)	112 (74.8)	0.734 (73.5)
Carnac (M)	448	294	259 (57.8)	18 (93.5)	0.735 (75.7)
Carnac (M) + RNAfold-C	448	471	337 (75.2)	110 (75.4)	0.753 (75.3)

**Table 8:** *E. coli *LSU rRNA is approximately 3350 nucleotides in length. The longest member of our test-set. The highest ranked Sfold prediction is remarkably poor, resolving just 5.8% of the reference structure.

	***E. coli *LSU rRNA: Single Sequence Methods**				
Algorithm	number of bps in reference	number of bps in prediction	True Positives (% sensitivity)	False Positives (% selectivity)	Correlation (%)

RNAfold	839	906	435 (51.8)	430 (50.3)	0.510 (51.1)
Mfold (1)	839	883	458 (54.6)	383 (54.5)	0.545 (54.5)
Mfold (2)	839	892	480 (57.2)	364 (56.9)	0.570 (57.0)
Mfold (3)	839	889	454 (54.1)	392 (53.7)	0.539 (53.9)
Sfold (1)	839	903	49 (5.8)	811 (5.7)	0.057 (5.8)
Sfold (2)	839	878	432 (51.5)	411 (51.2)	0.513 (51.4)
Sfold (3)	839	882	384 (45.8)	463 (45.3)	0.455 (45.6)

**Table 9:** Pfold predictions on both the high and medium similarity data-sets underflow on *E. coli *LSU rRNA. RNAalifold and Carnac, however, produce reasonable results.

	***E. coli *LSU rRNA: Comparative Methods**				
Algorithm	number of bps in reference	number of bps in prediction	True Positives (% sensitivity)	False Positives (% selectivity)	Correlation (%)
Plan A: ClustalW Alignment					
RNAalifold (H)	794	879	627 (79.0)	195 (76.3)	0.776 (77.6)
RNAalifold (H) + RNAfold-C	794	871	629 (79.2)	185 (77.3)	0.782 (78.2)
RNAalifold (M)	819	721	614 (75.0)	53 (92.1)	0.831 (83.5)
RNAalifold (M) + RNAfold-C	819	790	691 (84.4)	78 (89.9)	0.871 (87.1)
Pfold (H)	794	0	0 (0.0)	0 (0.0)	0.000 (0.0)
Pfold (M)	819	0	0 (0.0)	0 (0.0)	0.000 (0.0)
ILM (H)	794	1048	389 (49.0)	602 (39.3)	0.438 (44.1)
ILM (M)	819	1161	560 (68.4)	405 (58.0)	0.630 (63.2)
ILM-pknot (H)	869	1048	272 (31.3)	759 (26.4)	0.287 (28.8)
ILM-pknot (M)	869	1161	377 (43.4)	629 (37.5)	0.403 (40.4)
Plan B: Unaligned sequences					
Carnac (H)	816	422	390 (47.8)	7 (98.2)	0.685 (73.0)
Carnac (H) + RNAfold-C	816	873	674 (82.6)	156 (81.2)	0.819 (81.9)
Carnac (M)	821	508	463 (56.4)	14 (97.1)	0.740 (76.7)
Carnac (M) + RNAfold-C	821	865	682 (83.1)	147 (82.3)	0.827 (82.7)
